# Khosta: A Genetic and Structural Point of View of the Forgotten Virus

**DOI:** 10.3390/idr15030031

**Published:** 2023-06-01

**Authors:** Fabio Scarpa, Elena Imperia, Alessandra Ciccozzi, Stefano Pascarella, Miriana Quaranta, Marta Giovanetti, Alessandra Borsetti, Nicola Petrosillo, Massimo Ciccozzi

**Affiliations:** 1 Department of Biomedical Sciences, University of Sassari, 07100 Sassari, Italy; 2 Unit of Medical Statistics and Molecular Epidemiology, University of Campus Bio-Medico, 00128 Rome, Italy; 3 Unit of Gastroenterology, Department of Medicine, University Campus Bio-Medico, 00128 Rome, Italy; 4 Department of Biochemical Sciences “A. Rossi Fanelli”, University of Rome “La Sapienza”, 00185 Rome, Italy; 5 Sciences and Technologies for Sustainable Development and One Health, University of Campus Bio-Medico of Rome, 00128 Rome, Italy; 6 Rene Rachou, Fundação Oswaldo Cruz, Belo Horizonte 30.190-009, Minas Gerais, Brazil; 7 National HIV/AIDS Research Center, Istituto Superiore di Sanità, 00161 Rome, Italy; 8 Infection Prevention and Control-Infectious Disease Service, Fondazione Policlinico Universitario Campus Bio-Medico, 00128 Rome, Italy

**Keywords:** bat SARS-like coronaviruses, SARS-CoV-like viruses, horseshoe bats, *Rhinolophus*, khosta viruses, sarbecovirus, coronavirus, epidemiology, spillover

## Abstract

Bats are well-known to be natural reservoirs of various zoonotic coronaviruses, which have caused outbreaks of severe acute respiratory syndrome (SARS) and the COVID-19 pandemic in 2002 and 2019, respectively. In late 2020, two new *Sarbecoviruses* were found in Russia, isolated in *Rhinolophus* bats, i.e., Khosta-1 in *R. ferrumequinum* and Khosta-2 in *R. hipposideros*. The potential danger associated with these new species of *Sarbecovirus* is that Khosta-2 has been found to interact with the same entry receptor as SARS-CoV-2. Our multidisciplinary approach in this study demonstrates that Khosta-1 and -2 currently appear to be not dangerous with low risk of spillover, as confirmed by prevalence data and by phylogenomic reconstruction. In addition, the interaction between Khosta-1 and -2 with ACE2 appears weak, and furin cleavage sites are absent. While the possibility of a spillover event cannot be entirely excluded, it is currently highly unlikely. This research further emphasizes the importance of assessing the zoonotic potential of widely distributed batborne CoV in order to monitor changes in genomic composition of viruses and prevent spillover events (if any).

## 1. Introduction

The Betacoronavirus group includes well-known animal viruses that have the potential to cause pandemics, such as Middle East respiratory syndrome-related coronavirus (MERS-CoV), SARS-CoV, and SARS-CoV-2 [1]. Rhinolophid bats (Rhinolophidae: *Rhinolophus*) are the primary but not the only natural reservoirs and sources of zoonotic coronaviruses (CoV), which caused severe acute respiratory syndrome (SARS) outbreaks in 2002 and the COVID-19 pandemic in 2019, respectively [2]. These viruses, including SARS-CoV and SARS-CoV-2, along with related viruses found in bats and other animals (SARS-like coronaviruses or SARS-CoV-like viruses), belong to the subgenus *Sarbecovirus* of the *Betacoronavirus* genus in the Coronaviridae family [2]. Rhinolophid bats are found throughout Asia, Europe, and North Africa, and SARS-CoV-like viruses circulate in multiple species of *Rhinolophus* bats. In East Asia, the Chinese rufous (*R. sinicus*), greater (*R. ferrumequinum*), intermediate (*R. affinis*), Malayan (*R. malayanus*), least (*R. pusillus*), and king (*R. rex*) horseshoe bats are of particular importance [2]. In Europe, SARS-CoV-like viruses have been found in the greater, lesser (*R. hipposideros*), Mediterranean (*R. euryale*), Mehely’s (*R. mehelyi*), and Blasius’ (*R. blasii*) horseshoe bats [2]. As the receptor-binding domain (RBD) contains all the information needed to engage with the host receptor, several authors (see, i.a., Seifert et al. [3]) have classified sarbecovirus RBDs into different clades based on sequence and functional data. These clades include Clade 1, identified in Asian bats (which contains no deletions and binds to the host receptor angiotensin-converting enzyme-2 - ACE2), Clade 2, identified in Asian bats (which contains 2 deletions and does not use ACE2), and Clade 3, which is widespread in African and European bats (which contains 1 deletion and can primarily infect using bat ACE2) [3,4,5,6,7,8,9,10,11,12]. Clade 2 sarbecoviruses cannot use ACE2 as an entry receptor, possibly because of the two characteristic deletions that occur within the receptor-binding motif (RBM) of RBD. Therefore, it has been suggested that Clade 2 sarbecoviruses may infect cells using an unidentified alternative receptor. Clade 3 viruses also have an impaired ability to use ACE2 receptor possibly because of the single deletion occurring within the RBM [3].

In late 2020, two sarbecoviruses belonging to the Clade 3 were discovered in *Rhinolophus* bats in Russia, i.e., Khosta-1 was found in *R. ferrumequinum*, and Khosta-2 was found in *R. hipposideros* [3]. These viruses use the spike glycoprotein to enter target cells after binding to a compatible host cell receptor. Unlike MERS-CoV, which uses dipeptidyl-peptidase 4 (DPP4) as the cell surface receptor, the lineage B viruses of the sarbecovirus subgenus SARS-CoV and SARS-CoV-2 are known to use ACE2 [1]. Virus spillover from animal reservoirs can cause public health crises and disrupt the world economy, so surveillance is crucial to anticipate future pandemics. In this context, we conducted multidisciplinary analyses to test the potential of the Khosta virus. First, we conducted a phylogenetic analysis of the new *Sarbecovirus* species to reconstruct their evolutionary path and identify the conditions that could lead to a new spillover event. The analyzed dataset (n = 185) included all whole *Sarbecovirus* genomes found in bat species available on the National Center for Biotechnology Information (NCBI) virus portal (available at https://www.ncbi.nlm.nih.gov/labs/virus/vssi/#/, accessed on 15 January 2023) plus two variants of SARS-CoV-1 and SARS-CoV2 viruses each, available on the Global Initiative on Sharing All Influenza Data (GISAID) portal (available at https://www.epicov.org/epi3/frontend#44f16d, accessed on 15 January 2023). Subsequently, we carried out a combined analysis of molecular dynamics and structure to investigate the complexes formed between ACE2 and the RBDs of Khosta-1 and -2.

## 2. Materials and Methods

### 2.1. Phylogenetic Analyses

To reconstruct the evolutionary path of the new *Sarbecovirus* species, the analyzed dataset was built including all Sarbecoviruses isolated in bat species available on the NCBI virus portal (available at https://www.ncbi.nlm.nih.gov/labs/virus/vssi/#/, accessed on 15 January 2023), as well as SARS-CoV-1 and SARS-CoV-2 viruses (two for each variant of concern–VOC–available on the GSAID portal at https://www.epicov.org/epi3/frontend#44f16d, accessed on 15 January 2023). More specifically, the dataset comprised a total of 185 whole genomes of Sarbecoviruses, bat SARS-like-CoV, BtRs and BtRf Beta-CoV, SARS-CoV1, and SARS-CoV-2 viruses.

Phylogenetic reconstruction was conducted according to Scarpa et al. [13]. Genomes were aligned using the L-INS-I algorithm implemented in Mafft 7.471 [14] and manually edited using the software Unipro UGENE v.35 [15]. The best probabilistic model of genome evolution was determined using the software jModeltest 2.1.1 [16] with a maximum likelihood optimized search. Phylogenomic relationship among lineages have been investigated using the software MrBayes 3.2.7 [17]. Two independent runs, each consisting of four Metropolis-coupled Markov-chain Monte Carlo (MCMCMC; one cold and three heated chains) simulations, were run simultaneously for 5,000,000 generations, with trees sampled every 1000 generations. The first 25% of the 10,000 sampled trees were discarded as burn-in. Nodes with a posterior probability greater than 0.95 were considered statistically supported. The phylogenetic tree was visualized and edited using the software FigTree 1.4.0 (available at http://tree.bio.ed.ac.uk/software/figtree/, accessed on 3 March 2023) and GIMP 2.8 (available at https://www.gimp.org/downloads/oldstable/, accessed on 6 March 2023), respectively.

### 2.2. Structural and Molecular Dynamics Analyses

Homology models of Khosta-1 and -2 spike RBDs and N-terminal domains (NTDs) were built with Modeller 10.3 [18] using as a template the SARS-CoV-2 RBD in the PDB structure 6M0J and the NTD in the coordinate set 7B62, respectively. Model structures were displayed and analyzed with the graphic program PyMOL [19]. FoldX 5.0 was applied to optimize the side-chain conformation of the obtained models using the function “RepairPDB” [20]. To sample the fluctuations of the side-chain conformations and interactions, 100 homology models of the RBD and NTD domains were built by Modeler. The Modeler refinement stage of the homology modeling produces alternative models differing for conformational details, among which are side-chain rotamers. Each model was optimized using the FoldX 5.0 “RepairPDB” function. Structural properties were calculated for all models to evaluate their average and standard error. Net charges were predicted using PROPKA3 [21] setting pH = 7.0 as reference pH, though not necessarily reflecting the physiological environment. Surface electrostatic potential was calculated with the program APBS [22] and displayed as a two-dimensional projection with the SURFMAP software [23]. SURFMAP implements a method of “molecular cartography” by means of which a protein three-dimensional surface can be projected onto a two-dimensional plane. In this way, the distribution of different physicochemical features over the protein surface can be analyzed and compared.

Model structures of complexes between ACE2 and Khosta-1 and -2 RBDs were built with Modeler using as a template the SARS-CoV-2 complex reported in PDB structure 6M0J. The homology modes of the Khosta complexes were optimized by molecular dynamics. Molecular dynamics was carried out with the program GROMACS 2020.1 [24] using the force field AMBER99SB-ILDN [25]. The complexes were solvated in a truncated octahedron box with transferable intermolecular potential 3P (TIP3P) water molecules and a 1.5 nm distance from the system to the box edge. The solvated system was neutralized and set to a concentration of 0.15 M NaCl. All the simulations were calculated in periodic boundary conditions. The system was minimized with the steepest descent minimizer until convergence, namely until no change in energy between successive steps was detected. After minimization, the system was subjected to 100 ps of NVT and 100 ps of NPT relaxation at 300 K with a modified Berendsen thermostat (time constant 1 ps). The LINCS algorithm was applied to constrain the bond lengths. Electrostatic forces were calculated with the particle-mesh Ewald method [26] using a grid spacing of 0.16 nm. A cutoff of 1.0 nm was set for short-range electrostatic and Van der Waals interactions. At the end of the relaxation, the models were minimized using the same protocol applied at the beginning of the procedure.

Interaction energy between the spike RBD and ACE2 was predicted with the command “AnalyseComplex” of the Foldx 5.0 suite and Molecular Mechanics/Generalized Born Surface Area (MM/GBSA) in the HawkDock server [27]. Foldx 5.0 uses an empirical force field that describes the different free energy terms, including electrostatic interactions, hydrogen bonds, desolvation, and van der Waals contacts. MM/GBSA calculates binding free energies for macromolecules by combining molecular mechanics calculations and continuum solvation methods. In silico alanine scanning of the residues at the interface between RBD and ACE2 was carried out using the method available at the web server DrugScorePPI [28]. The method is a fast and accurate computational approach to predict changes in the binding free energy when each residue at the subunit interface is in silico mutated into alanine. Residues that lost more than 1.0 kcal/mol upon mutation into alanine were considered hotspots.

## 3. Results 

**Figure 1:**
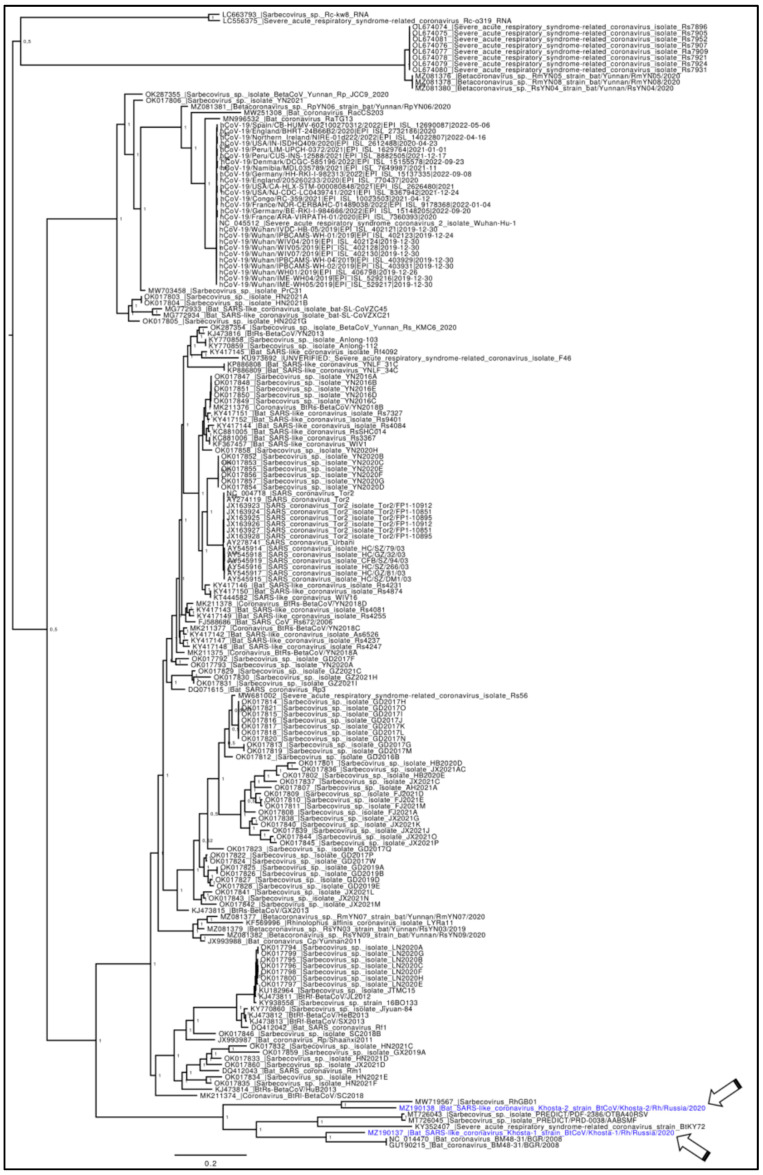
Phylogenomic reconstruction. The Bayesian phylogenetic tree, obtained by using a dataset including 185 whole genomes of Sarbecoviruses, viruses depict the phylogenetic relationship among lineages. Node support is expressed in posterior probabilities (PP). All of the main nodes are fully supported. Genomes of Khosta viruses are labeled in blue font and indicated with arrows.

The Bayesian phylogenetic tree, obtained by using a dataset including 185 complete genomes of Sarbecoviruses (Figure 1), together with bat SARS-like-CoV, BtRs and BtRf Beta-CoV, SARS-CoV1, and SARS-CoV2 viruses, shows clades with high support (posterior probabilities = 1). Overall, the tree shows a clear and statistically significant genetic structuring that is consistent with the viral strain taxonomic classification. After a midpoint rooting was applied, the tree indicated two distinct groups. The first one includes a heterogeneous group of SARS-related bats’ coronavirus comprising 13 sequences split into two statistically well-supported clusters, represented by genomes from Japan and from China collected from *Rhinolophus cornutus* (very common in Japan) and from *Rhinophus stheno*, *Rhinolophus malayanus*, and *Rhinolophus affinis* respectively. Samples in this clade were collected from 2013 to 2022. That clade, with respect to the main clade, is placed in a basal position, suggesting that it belongs to an ancient lineage.

The main clade is split into two groups. In the first one, there are 27 genomes of SARS-CoV-2 (isolated in human hosts) representative of different VOIs, six belonging to generic Sarbecovirus, one of Beta-CoV, and four of bat SARS-like-CoV.

The second group is formed by different clusters. The first cluster includes five sequences of Sarbecovirus from *Rhinolophus sinicus* and one of Beta-CoV from *Rhinolophus affinis*. Other clusters are composed of Sarbecovirus isolated from *Rhinolophus sinicus* in China, 11 of which in 2017; 4 in 2019 in August and September, respectively; 1 in 2020; and 4 in 2021. Within the main clade, a cluster is present comprising only sequences isolated from *Rhinolophus ferrumequinum*, nine of which from Sarbecovirus isolated in 2013, one in 2016, seven in 2020, and one BtRs-BetaCoV.

In the basal position of the second group, there are sequences of Khosta viruses: Khosta-1 from *Rhinolophus ferrumequinum* and Khosta-2 from *Rhinolophus hipposideros*, both isolated in Russia in 2020, collocated in two different clusters. It is interesting to note that Khosta-1 and Khosta-2 do not share a common ancestor and are not strictly closed from an evolutionary point of view. Indeed, Khosta-1 shows a sister species relationship with BtCoV/BM48-31/BGR/2008 isolated in Bulgaria in 2008 from *R. blasii*, with which it shares a common ancestor. Khosta-2 is evolutionarily close to RhGB01 isolated in the UK in 2020 from *R. hipposideros*.

**Figure 2:**
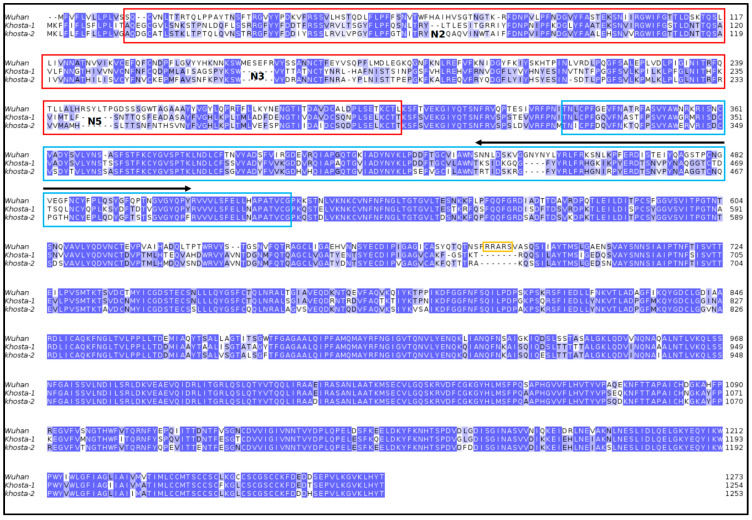
Sequence alignment among spikes from Khosta-1, Khosta-2, and SARS-CoV-2 Wuhan strain. NTD and RBD regions are evidenced by red and cyan boxes, respectively (see Table 1). The receptor-binding motif is denoted by a black arrow. The orange box indicates the neuropilin-1 binding site and the potential furin cleavage site, deleted in Khosta-1 and -2. Deleted loop regions are marked by N2, N3, and N5. Identically conserved sites are reported as white letters on blue background.

Sequences of Khosta-1 and -2 RBD and NTD were compared with the Wuhan SARS-CoV-2 counterparts. The NTD is less conserved than RBD (Table 1) both within Khosta viruses and with respect to Wuhan SARS-CoV-2 (Figure 2).

**Figure 3:**
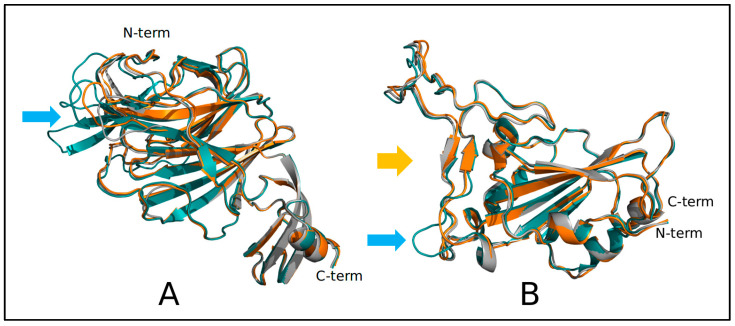
Superposition of (**A**) NBD and (**B**) RBD models of Khosta-1 (orange ribbon) and Khosta-2 (gray ribbon) with the Wuhan spike domains (deep teal ribbon) from the PDB structures 7B62 and 6M0J, respectively. The N- and C-terminal residues of chains are indicated. The orange arrow marks the interface with the ACE2 receptor, while cyan arrows highlight the indel regions.

This variability is reflected by the structural differences (Figure 2) between the modeled domains from Khosta and Wuhan viruses. The Khosta NTDs display several deletions at loops N2, N3, and N5 [29] that in SARS-CoV-2 are possibly involved in interaction with sialosides [30]. Khosta RBDs have a deletion in the receptor-binding region that possibly can weaken the interaction with ACE2 (Figure 3).

**Figure 4:**
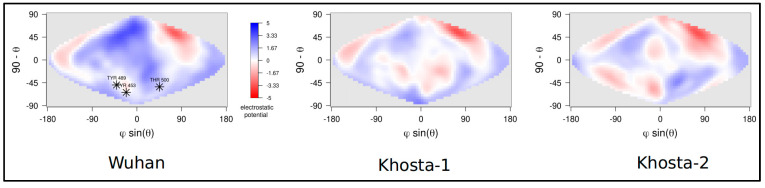
Projection on a two-dimensional map of the electrostatic potential surface of RBDs of the three viruses. Color scale is reported aside the Wuhan map. Electrostatic potential values are expressed as kT/e units. Map axes report the projected polar coordinates of the domains. Asterisks in Wuhan map indicate the position of the three residues T453, Y489, and T500 belonging to the interface with ACE2, belonging to the RBM.

Structural properties of the NTD and RBD domains have been compared to those of the corresponding portions of the Wuhan spike. The net charge, which approximates the intensity of the surface electrostatic potential, is significantly different (Table 2) except for Khosta-1 and Wuhan NTDs. A comparison of electrostatic surface potential maps of RBDs is reported in Figure 4. The comparison clearly shows that Khosta-1 and Khosta-2 RBDs have a less positive electrostatic potential. Moreover, the comparison of the three projections highlights not only the lower charge of Khosta viruses but also changes with the surface distribution of the potential in correspondence of RBM regions. These changes are caused also by the deletion occurring within the RBM in Khosta viruses.

**Figure 5:**
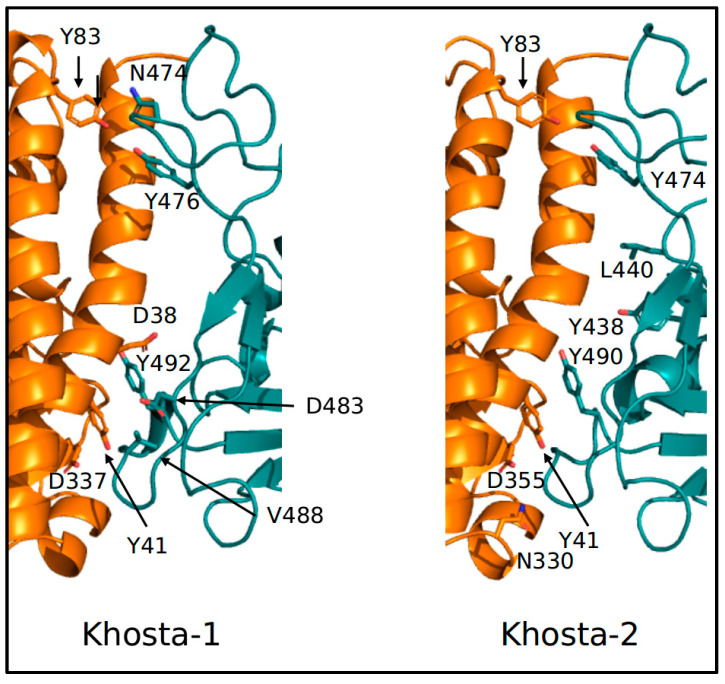
Hotspot residues at the predicted interface between ACE2 and Khosta RBDs. ACE2 and RBD are displayed as orange and deep teal ribbons, respectively. Hotspot residues are displayed with stick modes and labeled.

Accordingly, prediction of interaction energies between ACE2 and the RBDs from the three viruses suggests that binding of Khosta-1 and -2 RBD to the receptor is weaker that in the case of Wuhan virus (Table 3). Moreover, the complex ACE2-RBD of Wuhan SARS-CoV-2 (PDB-ID 6M0J) is predicted to have six hotspot residues in RBD (Y449, L455, N487, Y489, N501, Y505) and five in ACE2 (D38, Y41, Y83, K353, D355). Khosta-1 has five and four residues, respectively, while Khosta-2 has four for both interacting domains (Figure 5).

## 4. Discussion

The current pandemic of COVID-19, caused by the SARS-CoV2, has been generated by a spillover event of *Sarbecovirus* from animals to humans [31]. *Sarbecovirus* is a subgenus of *Betacoronavirus* which is the primary cause of respiratory syndrome. It is composed of two main viral strains, SARS-CoV-1 and the SARS-CoV-2. The last discovered *Sarbecoviruses*, Khosta-1 (isolated in *Rhinolophus ferrumequinum*) and Khosta-2 (isolated in *Rhinolophus hipposideros*) [3], have recently caused several concerns. The potential problem linked to these new species of *Sarbecovirus* is that Khosta-2 has been shown to interact with the same entry receptor as SARS-CoV-2 [3]. In this context, also considering the importance of bats in the case of spillover, the case of Khosta-2 requires a deep integrative approach in order to obtain a clear perspective of the chance of spillover. One of the most striking results is given by the lack of consistency between our phylogenomic reconstruction and the experimental classification performed on sequences and functional data for the published *Sarbecovirus* RBDs, which assumes the occurrence of 3/4 clades (see, i.a., Seifert et al. [3]). Indeed, the most inclusive clade is composed of viruses belonging to Clades 1 to 3, in accordance with the classification proposed experimentally on sequence and functional data [3]. It should be noted that the internal relationship among clades is not consistent with previous findings based on the classification of sarbecovirus RBDs. This discrepancy may be probably due to the genome-based approach that includes within the dataset a higher number of molecular characteristics that provide an information based on the genetic identity (which may be affected by genetic drift) and may be not strictly related to functional features. In addition, it should be noted that functional classification may reflect homoplasy due to an adaptive convergence occurring during the viral evolutionary path, while the evolutionary distances are based on the neutral theory of molecular evolution [32]. However, discrepancies do not present implications for public health or for surveillance, providing only a further confirmation that the genome-based multidisciplinary approach allows a complete point of view.

In the case of Khosta, the most striking results are given by the genetic distance between the two species of Khosta virus. Indeed, our phylogenomic reconstruction does not support their reciprocal monophyletic condition as previously supposed [3]. Khosta-1 is evolutionarily close to BtCoV/BM48-31/BGR/2008 isolated in Bulgaria in 2008 from *R. blasii* (with which it shares a common ancestor), while Khosta-2 is evolutionarily close to RhGB01 isolated in the UK in 2020 from *R. hipposideros*. It should be noted that although the capability of Khosta-2 to interact with the same entry receptor as SARS-CoV-2, genomes of Khosta are placed in a well-supported different clade than SARS-CoV-2 and other pathogenic *Sarbecoviruses*. This evolutionary distance suggests a current low risk of spillover of Khosta from bats to humans. This finding is not so obvious but is not unusual. Indeed, sometimes viruses (or pathogens in general) are released from wild animals to domestic ones or humans, and just as often this occurs in a cryptic way. Very often, viruses present a rapid initial adaptation in their hosts and can improve the fit over time, staying silent for a long time without causing disease (see e.g., HIV-1 infections [33]) or without spillover.

For instance, in recent times within the family *Coronaviridae*, the *Alphacoronavirus*, named swine acute diarrhea syndrome coronavirus (SADS-CoV), was identified as the cause of an outbreak in the pig population in the southeast of China (province of Guangdong) in early 2017 and rapidly spread in several farms, persisting for a few months. In the beginning, this caused a general alert for human health because the *Alphacoronaviruses* demonstrated higher odds of host-switching than *Betacoronaviruses*, which in turn are more linked to the phylogenetic distance among hosts [34]. Indeed, *Alphacoronaviruses* are known for not having a good proofreader; thus, many errors accumulate in the replication phase, causing a quick evolutionary and mutation rate. After several years, it appears that spillover from pigs to humans did not occur. Our results suggest a similar condition for Khosta viruses, which currently appear as an evolutionary blind background in the phylogenomic tree, suggesting the absence of an imminent threat.

In addition, it should be noted that interaction of Khosta-1 and -2 with ACE2 seems to be weaker than Wuhan spike neuropilin-1 interaction, and furin cleavage sites are missing. In this context, it is interesting to note that in Wuhan SARS-CoV-2 loops are deemed to interact with sialosides, and in Khosta NTDs several loops are deleted. All of these findings further confirm the need for constant and uninterrupted genome-based monitoring. Indeed, continuous monitoring of pathogens, with the aim of improving public health, remains the best tool for epidemiological surveillance of viruses that can potentially perform a spillover event.

However, it should be highlighted that, although genetic monitoring is always the first choice in order to be prepared in case of a new outbreak or a new epidemic, a spillover event cannot be predicted. Indeed, a spillover event can occur due to a variety of factors, such as changes in environmental conditions and contact between different species in a concentrated area. Accordingly, when a spillover event occurs, the disease may have devastating effects on the new host species, particularly if they have not developed immunity to it. The genetic monitoring provides information on the expansion capabilities of the virus, and although spillover is an abrupt and unpredictable event, the viral population with a low level of genetic variability and slow evolutionary rate presents a low chance [35].

Regarding unpredictable events, the recombination should not be underestimated. Indeed, together with reassortment (which concerns influenza viruses (see Mugosa et al. [36]), recombination is one of the main promoters of the increasing of variability in RNA viruses [37]. It is very common and widely documented in the family Coronaviridae. For instance, a recombination event occurred in the *Alphacoronavirus* SADS-CoV [34], such as in SARS-CoV-2 in recent times, generating the lineage XBB nicknamed *Gryphon* [38]. Of course, it cannot be excluded that Khosta takes part as an acceptor or donor in a recombination event (especially since it is very frequent in the animal world), but it should be considered that involved strains must be very common with high prevalence in order to infect the same host simultaneously.

Although data reported here are fully statistically supported and were proved to have high levels of repeatability, it should be noted that they are based on in silico simulation and theoretical models and not directly on experimental results and may be affected by bias caused by the lack of important strains in the dataset.

## 5. Conclusions

In conclusion, the present research further underlines the significance of evaluating the zoonotic potential of widely prevalent batborne CoV in order to limit spillover events. Indeed, spillover events can also have significant implications for human health, as diseases that were previously confined to animal populations can spread to humans. This can have serious consequences, particularly if the new disease is especially virulent or resistant to treatment. As such, understanding and predicting spillover events is crucial for protecting both animal and human health. In general, bats are well-known to be the natural reservoirs of a variety of zoonotic coronaviruses, which can cause epidemic outbreaks of severe acute respiratory syndrome. However, Khosta viruses in their current state do not represent a threat to public health. Indeed, both Khosta-1 and Khosta-2 present a low level of genetic variability and relatively slow mutation rates, which have also been confirmed by their position in the phylogenomic tree, where they are placed in long branches, appearing as lineages without descendants with evolutionary features typical of a blind background. Of course, this result must not be misconstrued as a reason to let down our guard against viruses (or pathogens in general), and the monitoring must continue uninterrupted. Indeed, one of the most important conclusions from the current COVID-19 pandemic is the need for extensive genome-based monitoring of all viruses and lineages (and their descendants) in order to identify and predict important changes in their genomic composition that may lead to potentially serious events.

## Figures and Tables

**Table 1:** Sequence comparison of RBD and NTD from Khosta-1, Khosta-2, and Wuhan SARS-CoV-2. Numbers represent the percentage sequence identity for NTD and RBD. Boldfaced numbers refer to the comparison of NTDs and the others RBDs.

	Khosta-1	Khosta-2	Wuhan
Khosta-1	-	64	44
Khosta-2	79	-	46
Wuhan	70	68	-

**Table 2:** Comparison between structural properties of the NTD and RBD domains. Numbers indicate the predicted net charge (that approximates the intensity of the surface electrostatic potential) at pH 7.0. ^a^ Mean ± standard error over 100 homology models; ^b^ Net charge of 6M0j RBD and 7B62 NTD.

	Khosta-1 ^a^	Khosta-2 ^a^	Wuhan ^b^
RBD	0.08 ± 0.01	0.79 ± 0.01	2.13
NTD	1.14 ± 0.05	4.89 ± 004	1.50

**Table 3:** Prediction of interaction energies between ACE2 and the RBDs from the Khosta-1, Khosta-2, and Wuhan SARS-CoV-2. Predicted interaction energies are expressed in kcal/mol.

Method	Khosta-1	Khosta-2	Wuhan
FoldX 5.0	−12.00	−9.40	−16.88
Mm/GBSA	−42.14	−48.81	−65.45

## Data Availability

Genomes analyzed in the present study were taken from the GSAID database (available at https://gisaid.org/, accessed on 15 January 2023) and from NCBI virus portal (available at https://www.ncbi.nlm.nih.gov/labs/virus/vssi/#/, accessed on 15 January 2023).
